# Qualifications for Admission to the Register

**Published:** 1916-06-10

**Authors:** 


### QUALIFICATIONS FOR ADMISSION TO THE REGISTER.

The leaflet referred to in Mr. Stanley's letter, after setting out in the first clause five of the objects of the College, continues as follows:—

2. The first Council of the College has been constituted by nomination and co-optation, but in 1918 and every year afterwards one-third of its members retires by rotation, and vacancies are filled by the votes of nurses on the Register who are also members of the College. Hence the Council has decided that, though some months must necessarily elapse before they will be in a position to lay before the Consultative Board their proposals with regard to the curriculum and the recognition of training schools, no time should be lost in informing the nursing profession of the general conditions under which they are prepared to admit trained nurses already in practice to the Register of the College. The Regulations are as follows:—

3. Applicants are required—

(a) To be at least twenty-one years of age.

(b) To be of good character.

(c) To hold a certificate or certificates of three years' training in a nurse-training school or schools recognised by the Council for the purpose of admitting practising nurses to the Register of the College; or

(d) To hold a certificate of not less than two years' training in a nurse-training school recognised by the Council for the purpose of admitting practising nurses to the Register, followed by at least two years' *bona fide* practice as a nurse; or

(e) To produce evidence of training to the satisfaction of the Council, having regard to the date at which the training was taken, followed by at least five years' *bona fide* practice as a nurse.

4. The fee for registration and membership has been fixed at £1, but when a practising nurse is already a member of an Association where the conditions of membership correspond with the above general regulations of the College, the Registration Committee may recommend her for membership of the College at a reduced or nominal fee.

5. The Council has already resolved to promote a Bill in Parliament for the Registration of Nurses and the protection of the title of Registered Nurse. Under the Bill it is the duty of the College of Nursing to form and keep the legal Register, and "the Register already formed by the College of Nursing shall be the first Register under this Act." It is, therefore, of the utmost importance that, when the Bill comes before Parliament, the Register already formed by the College should be found to include a great majority of all trained nurses now in practice, and to attain this result the Council appeals with confidence to the active support of the nursing profession and of the authorities of the nurse-training schools throughout the country.

[It will be seen on reference to the Notes in "Round the Hospitals," on page 241, the extent to which we have forecast some of the difficulties which the College has initially to face and the proposals for their solution, as set out in the above document.—Ed. The Hospital.]

